# Platelet/lymphocyte ratio for prediction of no-reflow phenomenon in ST-elevation myocardial infarction managed with primary percutaneous coronary intervention

**DOI:** 10.18053/jctres.06.202001.004

**Published:** 2020-07-08

**Authors:** Hala Mahfouz Badran, Ahmed Abdel Fatah, Ghada Soltan

**Affiliations:** ^1^Department of Cardiology, Menoufia University, Egypt; ^2^National Heart Institute, Egypt

**Keywords:** platelet/lymphocyte ratio, primary percutaneous coronary intervention, ST-segment elevation myocardial infarction

## Abstract

**Background:**

Coronary no-reflow phenomenon in ST-segment elevation myocardial infarction (STEMI) is associated with a poor clinical outcome. Although its pathophysiology is not fully understood, a deregulated systemic inflammatory response plays an important role. We aimed to explore the relationship between platelet\lymphocyte ratio (PLR) and no-reflow in patients with acute STEMI who were treated with a primary percutaneous coronary intervention (PPCI).

**Methods:**

A total of 200 patients with STEMI undergoing PPCI were included in the study. Transthoracic echocardiographic examination was performed to assess left ventricular (LV) ejection fraction (EF) and wall motion score index. Blood samples were assayed for platelet and lymphocyte count before PPCI. No-reflow was defined as coronary blood flow thrombolysis in myocardial infarction grade ≤II.

**Results:**

No-reflow was observed in 58 (29%) of STEMI patients following PPCI. PLR was significantly higher in hypertensive patients compared to normotensive patients (144.7±91.6 vs. 109.1±47.1, respectively, *P*<0.001) and in the no-reflow group compared to the normal reflow group (214±93 vs. 101.6±51.3, respectively, *P*<0.0001). Logistic regression analysis revealed that PLR (β: 0.485, 95% CI: −0.006-0.001, *P*<0.002) and LV EF (β: 0.272, 95% CI: 0.009-0.034, *P*<0.001) were independent predictors of no-reflow after PPCI.

**Conclusion:**

Pre-procedural increase in PLR is predictive of the no-reflow phenomenon following PPCI in STEMI patients.

**Relevance for Patients:**

No reflow phenomenon is an unfavorable complication following PPCI in patients with acute STEMI. High pre-procedural PLR is an independent predictor of reperfusion failure and helps to identify patients who require prophylactic treatment.

## 1. Introduction

In acute ST-segment elevation myocardial infarction (STEMI), primary percutaneous coronary intervention (PPCI), and stent implantation are the first choice of treatment [1,2]. However, earlier studies demonstrated a high incidence of coronary slow/no-reflow in 1-40% of the patients that may be associated with stoppage of myocardial perfusion restoration, whereby patients continued to suffer from the severe impairment [3,4]

No reflow is recorded in large registries based on thrombolysis in myocardial infarction (TIMI) flow grade, myocardial blush grade, ST resolution [5], myocardial contrast echocardiography, and cardiac magnetic resonance imaging that assessed microcirculatory dysfunction [6].

Several hypotheses have been formulated to describe the pathogenesis of no-reflow, including distal microembolization of thrombus fragments, swelling of endothelial cells caused by ischemia-reperfusion injury, and microvascular spasm [7-10]. A large number of studies have been carried out to investigate the predictors of slow/no-reflow phenomenon and the results showed that thrombosis burden, reperfusion time, and inflammatory factors are implicated [11-16].

Platelet activation plays a central role in the initiation and progression of atherosclerosis [17], and increased platelet activation is associated with major adverse cardiovascular consequences [18-20]. On the other hand, a low blood lymphocyte count has been shown to be related to worse cardiovascular outcomes in patients with coronary artery disease. The aim of this study was to explore the relationship between the platelet/lymphocyte ratio (PLR) and post-intervention TIMI flow in STEMI patients who have undergone PPCI.

## 2. Patients and Methods

This cross-sectional observational study was conducted on patients presented with STEMI and treated with PPCI between December 2017 and August 2019. We investigated 200 consecutive patients presented in two tertiary referral centers.

Patients with one or more of the following criteria were excluded from the study: Prior acute coronary syndrome, non-STEMI, unstable angina, STEMI duration more than 12 h, cardiogenic shock, treatment with thrombolytic therapy in the previous 24 h, estimated glomerular filtration rate <60 mL/min/1.73 m^2^ or renal dialysis, active systemic inflammatory diseases, or active treatment for specific conditions (including allergy, asthma, autoimmune diseases, glomerulonephritis, hepatitis, inflammatory bowel disease, and known malignancy).

All patients were reviewed for their risk profile, including smoking, hypertension, diabetes, dyslipidemia, and family history. Twelve leads electrocardiography (ECG), conventional echocardiography for evaluation of left ventricular (LV) function using ejection fraction (EF%), and wall motion score index (WMSI) were performed.

### 2.1. Blood analysis

Routine laboratory investigations included platelet, lymphocyte count, hemoglobin (HB), serum creatinine, cardiac biomarkers including troponin and creatine kinase myocardial band (CK-MB). Venous blood samples were drawn from antecubital veins immediately after patient evaluation and ECG recording.

Whole blood was analyzed on a Sysmex K-1000 and Sysmex XN-10 Automated Hematology Analyzer (Sysmex Corporation, Kobe, Japan) immediately following blood sampling. Whole blood was collected in ethylene diamine tetraacetic acid containers.

Before PPCI, all patients received 300 mg aspirin and 600 mg clopidogrel at the time of diagnosis before the intervention, and an intravenous bolus of unfractionated heparin 40-70 U/kg to achieve an activated clotting time of 200-250 s during the procedure. Coronary angiography was performed using standard techniques (Siemens Axiom Artis zee 2011 standard) encompassing a femoral approach with a 6-French guiding catheter. Direct stenting, balloon pre-dilatation, and the use of balloon pre-dilatation or post-dilatation, the type of stents, the use of tirofiban, and thrombus aspiration were at the operator’s discretion.

The TIMI flow grade was evaluated by two independent, experienced interventional cardiologists using quantitative cardiovascular angiographic software. The TIMI flow was assessed where TIMI 0 was defined as no antegrade filling of the culprit vessel, TIMI I was defined as sluggish filling and evacuation of the culprit vessel, TIMI II was defined as normal filling with sluggish evacuation, and TIMI III was defined as normal filling with normal evacuation.

Angiographic slow/no-reflow during PCI was defined as TIMI flow grade of ≤II during the procedure without evidence of dissection, residual stenosis, distal embolism, or vasospasm.

The study patients were divided into two groups based on the post-intervention infarct-related artery flow: The normal-reflow group included patients with post-intervention TIMI flow grade of III and the no-reflow group included patients with post-intervention TIMI flow grade 0, I, and II.

### 2.2. Statistical analysis

Data were collected, coded, revised, and entered into the Statistical Package for the Social Science (SPSS). Data were presented as mean±SD for continuous data and as number (%) for categorical data. Logistic regression analysis was used to assess the risk factors for coronary flow. The confidence interval was set to 95% and the margin of error accepted was set to 5%. *P*≤0.05 was considered statistically significant.

## 3. Results

**Figure 1:**
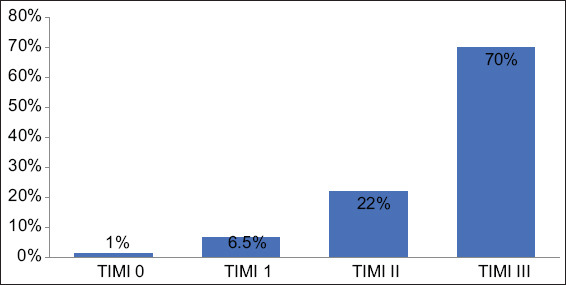
Coronary flow profile following primary percutaneous coronary intervention.

Two hundred patients were enrolled in the study, their mean age was 52.9±11.1, body mass index was 27.6±2.5, 160 (80%) patients were male, 118 (59%) were smokers, 88 (44%) were diabetic, 102 (51%) were hypertensive, 35 (17.5%) were obese, and 41 (20.5%) were dyslipidemic. Twenty-eight patients (14%) had a positive family history, 123 (61.5%) had anterior STEMI, 75 (37.5%) had inferior STEMI, and 2 (1%) had lateral STEMI. Twelve (6%) patients had no risk factors, 52 (26%) had one risk factor, 69 (34.5%) had two risk factors, 51 (25.5%) had three risk factors, and 16 (8%) had four risk factors. The mean CK-MB was 104±67, the mean troponin was 7.8±3.2, the PLR was 14.22±11.2, the EF was 46.5%±7.7%, and the WMSI was 1.2±0.1 (Figure 1). Demographic and clinical characteristics of studied groups according to TIMI flow are presented in Table 1.

**Table 1:** Patients characteristics and risk factors.

	TIMI 0-II (n=58)	TIMI III (n=142)	*P*-value
Male (%)	49 (84.5%)	111 (78.2%)	0.2
Female (%)	9 (15.5%)	31 (21.8%)	
Obese (%)	9 (15.5%)	26 (18.3%)	0.84
Diabetic (%)	26 (44.8%)	62 (43.7%)	0.92
HTN (%)	31 (53.4%)	71 (50%)	0.87
Dyslipidemia (%)	7 (12.1%)	34 (23.9%)	0.23
Smokers (%)	37 (63.8%)	81 (57%)	0.22
+ ve family history (%)	4 (6.8%)	24 (16.9%)	0.11
Troponin (ng/mL)	8.2±3	5.1±2.4	0.07
CK-MB (IU/L)	195±35	104±24	0.01
Ejection fraction (%)	40±6	56±4	0.03
Platelet (×10^3^/μL)	345±114	228±84	0.0001
Lymphocyte (×10^3^/μL)	1.73±0.5	2.2±0.9	0.02
Platelet/lymphocyte ratio	199.4±52	102±53	0.001
Infarction site			
Anterior (%)	44 (35.7%)	79 (64.2%)	0.07
Lateral (%)	1 (50%)	1 (50%)	
Inferior (%)	13 (17.3%)	62 (82.7%)	
Left anterior descending (%)	44 (35.7%)	79 (64.2%)	0.4
Left circumflex (%)	3 (14.3%)	18 (85.7%)	
Obtuse marginal 1 (%)	1 (33.3%)	2 (66.7%)	
Obtuse marginal 3 (%)	0 (0.0%)	1 (100%)	
Right coronary artery (%)	10 (19.2%)	42 (80.8%)	

PPCI to the culprit vessel was performed; 123 patients (61.5%) had percutaneous coronary intervention (PCI) to left anterior descending with 140 (70%) drug-eluting stents (DESs), 21 patients (10.5%) had PCI to left circumflex with 21 (10.5%) DESs, three patients (1.5%) had PCI to OM1 with 3 (1.5%) DESs, one patient (0.5%) had PCI to OM3 with 1 (0.5%) DES, and 52 (20%) patients had PCI to RCA with 52 (20%) DESs.

Coronary flow was graded using TIMI flow and showed that 2 (1%) patients had TIMI 0, 13 (6.5%) patients had TIMI I, 44 (22%) patients had TIMI II, and 141 (70.5%) patients had TIMI III.

We studied the relationship between platelet, lymphocyte, and PLR and risk factors (clinical and angiographic findings) after successful PCI. The platelet count was significantly higher in hypertensive patients compared to non-hypertensive patients; 271.5±111 versus 237.2±87.8, *P*<0.017, respectively, and in the no-reflow group compared to normal reflow group; 345±114 versus 228±84, *P*<0.0001, respectively. The lymphocyte count was significantly lower in the no-reflow group compared to the reflow group, 17.3±5% versus 25±9, *P*<0.0001. PLR was significantly elevated in hypertensive patients compared to non-hypertensive patients: 14.5±9.2 versus 10.9±4.7, *P*<0.001, respectively, and in the no-reflow group compared to the normal reflow group: 23.7±8 versus 9.1±5.3, *P*<0.001, respectively (Table 2).

**Table 2:** Platelet, lymphocytes, and PLR values in the study cohort.

	No.	Platelet	*P*-value	Lymphocyte	*P*-value	PLR	*P*-value
Male	160	248.7±98.7	0.9	2.3±0.9	0.8	125±73	0.4
Female	40	278.9±110		2.3±0.9		137±85	
Obese	35	269.7±110	0.3	2.4±0.9	0.5	129±91	0.8
Non-obese	165	251.5±99.8		2.2±0.9		127±72	
Smokers	118	252±97	0.6	2.3±0.9	0.4	125±75	0.6
Non-smokers	82	258.6±108.6		2.2±0.8		131±75	
Hypertensive	102	271.5±111	0.01	2.2±0.8	0.9	145±92	0.001
Non-hypertensive	98	237.2±87.8		2.3±0.9		109±47	
Diabetic	88	262.9±108	0.3	2.2±1.0	0.9	134±86	0.3
Non-diabetic	112	248.3±96.2		2.3±0.8		122±65	
Dyslipidemia	41	237.7±78.9	0.2	2.2±0.8		123±58	0.6
No dyslipidemia	159	259.1±106		2.3±0.9	0.4	128±79	
Family history	28	244.6±101	0.6	2.6±0.9	0.06	104±59	0.8
No family history	172	256.3±102		2.2±0.8		131±77	
TIMI 0	3	373.6±126	0.00001	1.7±0.5	0.00001	285±124	0.00001
TIMI I	10	360.6±115		1.6±0.4		258±114	
TIMI II	45	302.8±103.5		1.8±0.6		188±65	
TIMI III	142	228.6±84.6		2.2±0.9		102±53	
No. of risk factors							0.5
0	12	266.42±94.7	0.5	2.3±0.8	0.9	122±39	
1	52	233.9±93.9		2.3±0.8		112±59	
2	69	265.7±107.1		2.3±1.0		134±79	
3	51	256.0±104.2		2.2±0.7		135±97	
4	16	261.8±101.3		2.4±1.0		127±76	
TVR: LAD	123	253.8±103.3	0.7	2.2±0.8	0.07	130±73.31	0.6
LCX	25	270.4±93.7		2.5±0.8		122±76.4	
RCA	52	249.3±102.7		2.4±1.1		121±79.8	

TVR: Target vessel revascularization, LAD: Left anterior descending, LCX: Left circumflex, RCA: Right coronary artery

Pearson correlation coefficient was used to examine the relationship between platelet, lymphocyte, and PLR and the patients’ clinical and angiographic findings (Table 2). From all clinical and angiographic data, platelet counts showed a direct correlation to CK-MB (*P*<0.004) and TIMI flow (*P*<0.000), respectively. The total lymphocytic count was inversely correlated to TIMI flow (*P*<0.000) and directly correlated with HB (*P*<0.001). PLR ratio showed a direct correlation to CK-MB (*P*<0.006) and TIMI flow (*P*<0.0001) (Table 3).

**Table 3:** Correlation of PLR with patient’s characteristics.

	Platelet	Lymphocyte	PLR
Age (years)			
r	−0.113	−0.100	−0.023
p	0.111	0.158	0.741
BMI (kg/m^2^)			
r	0.083	0.034	0.048
p	0.240	0.627	0.495
EF (%)			
r	−0.052	−0.002	−0.034
p	0.467	0.982	0.636
Troponin (ng/mL)			
r	0.232	−0.047	0.180
P	0.005	0.331	0.006
CK-MB (IU/L)			
r	0.202	−0.056	0.193
p	0.004	0.431	0.006
WMSI			
r	0.079	−0.057	0.091
p	0.267	0.419	0.198
Cr (mg/dL)			
r	−0.002	−0.132	0.119
p	0.980	0.062	0.092
TIMI flow			
r	−0.434	0.339	v0.599
p	0.000	0.000	0.000
HB (g/dL)			
r	0.009	0.228	−0.100
p	0.902	0.001	0.158
Gensini score			
r	−0.018	−0.042	0.013
p	0.799	0.553	0.859

EF: Ejection fraction, WMSI: Wall motion score index, G: Gensini score, Cr: Creatinine, HB: Hemoglobin

**Figure 2:**
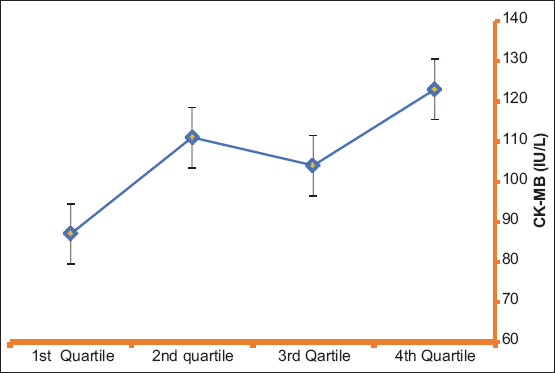
Creatine kinase myocardial band levels stratified per platelet\lymphocyte ratio quartile.

Multivariate logistic regression analysis was employed to identify the independent predictors of TIMI flow in STEMI patients following PPCI. PLR (β: −0.485, 95% CI: −0.006-0.001, *P*<0.002) and EF % (β: 3.407, 95% CI: 0.009-0.034, *P*<0.001) were independent predictors of TIMI flow in STEMI patient after PPCI (Table 4 and Figure 2).

**Table 4:** Predictors of TIMI flow in STEMI patients after primary PCI.

	B	SE	β	*t*	*P*-value	95% CI	

						Lower	Upper
HTN	0.039	0.071	0.032	0.551	0.582	−0.100	0.178
Platelets (/mm^3^)	0.000	0.001	−0.073	−0.564	0.573	−0.002	0.001
Lymphocyte (%)	0.068	0.069	0.100	0.980	0.328	−0.069	0.204
PLR	−0.004	0.001	−0.485	−3.186	0.002	−0.006	−0.001
BMI (kg/m^2^)	−0.021	0.014	−0.087	−1.509	0.133	−0.047	0.006
EF%	0.021	0.006	0.272	3.407	0.001	0.009	0.034
WMSI	0.260	0.320	0.065	0.812	0.418	−0.371	0.891
Cr (mg/dl)	−0.056	0.118	−0.028	−0.479	0.633	−0.289	0.176

## 4. Discussion

In the present study, PLR was an independent predictor of no-reflow in STEMI patients treated with PPCI. PLR was directly correlated to cardiac enzyme level (CK-MB) and showed higher values in hypertensive patients. STEMI patients that had regained normal coronary flow (TIMI III) had considerably lower platelet count and PLR but higher lymphocytic count compared to patients with slow or no flow.

Several studies have shown a relationship between the no-reflow phenomenon and increased inflammatory activity and proposed the PLR as a surrogate pro-thrombotic and inflammatory marker [21-23]. Our findings confirm the relation between PLR and the occurrence of no-reflow as a post-procedural complication following PPCI.

Although the pathophysiology of no-reflow has not been fully elucidated, its cause appears to be multifactorial. These factors include reperfusion injury from neutrophil aggregation, microvascular leukocytes, platelets plugging, complex interactions between neutrophils and platelets induced by the inflammatory process, distal embolization of culprit lesion, endothelial damage, and the production of reactive oxygen species [24-26].

PLR first gained attention in cancer patients as a marker for prognosis [27,28], after which it received growing interest with respect to its usefulness as a prognostic marker in cardiovascular medicine. The proposed mechanism of platelet involvement is platelet activation as a pivotal step of the inflammatory response in CAD and cardiovascular events [29,30]. During inflammation, a variety of inflammatory mediators (e.g., interleukin [IL]-1, IL-3, and IL-6) are released that stimulate megakaryocytes to proliferate and increase platelet levels in the circulation [31]. Activated platelets promote a pro-inflammatory environment by secreting cytokines and coagulation factors and they play a key role in the progression of atherosclerosis [32]. On the other hand, lymphocytes are responsible for providing a regulatory and quiescent pathway of inflammation [31,33].

Early risk stratification to detect patients at high risk of angiographic no-reflow is very important for its prevention in addition to early treatment using pharmacological and/or interventional strategy.

A recent analysis of eight pooled cohorts with a total of 6627 patients with acute coronary syndrome demonstrated that high PLR (>150) doubles the risk of in-hospital, all-cause, and cardiovascular mortality (pooled relative risk, 2.15; 95% CI, 1.73-2.67, 1.95, and 1.30-2.91, respectively) [34].

Prior studies demonstrated the association between PLR and cardiovascular events. Azabet *et al*. [35] showed higher PLR independently predicted 4-year mortality in non-STEMI patients, while Osadnik *et al*. [36] demonstrated the predictive value of PLR in patients with stable CAD undergoing PCI and stent implantation. Cho *et al*. [37] investigated the prognostic value of PLR and neutrophil-lymphocyte ratio (NLR) in patients without STEMI undergoing elective PCI with drug-eluting stents and showed PLR and NLR, alone and in combination, predicted long-term major adverse cardiovascular events.

In our study, we investigated 200 patients presented with STEMI and no previous history of the acute coronary syndrome. The patients were subjected to PPCI within 12 h of presentation and divided into two groups based on the TIMI flow grade: Normal reflow in 71% of patients with TIMI flow grade III, while no-reflow developed in 29% of patients with TIMI flow grade ≤II. Patients with no-reflow were predominantly male, hypertensive, had higher WMSI, PLR, and lower EF compared to those with normal flow. In contrast, other risk factors did not differ between groups. Using logistic regression analysis, PLR and EF were independent predictors of no-reflow after PPCI.

Similarly, Kurtul *et al*. [31] investigated 520 patients with acute STEMI and reported a lower rate of no-reflow (22% of patients). These patients were older than the patients who had regained normal coronary flow. PLR on the admission of ≥126 predicted the angiographic no-reflow with 73% sensitivity and 71% specificity. Moreover, PLR and stent length were independent predictors of no-reflow following PPCI.

Taken together, with the published data, the current study raises the potential role of inflammation theory and underscores the value of inflammatory markers in the pathophysiology of coronary circulation and no-reflow phenomenon. However, further studies are required to explain the exact mechanism of PLR in the pathogenesis of this phenomenon.

### 4.1. Study limitations

Our study has some limitations. First, the small sample size and there was no patient follow-up to examine the occurrence of adverse cardiac events and to explore the relationship between these cardiac events and PLR. Second, other inflammatory markers such as endothelin 1 and thromboxane A2 were not measured. Third, the incidence of no-reflow during PCI ranged widely from 1 to 41% [3-8]. While there are other studies that also demonstrate high rates of no-reflow, most large contemporary studies do not. The possible explanation for this difference might lie in the clinical and procedural characteristics and the application of a standardized definition of no-reflow. Although no-reflow is commonly recognized as transient, angiographically visible flow impairment despite epicardial coronary patency, other studies have included more liberal definitions, such as a failure to achieve TIMI III flow at the end of the procedure or decreased myocardial flow after PCI as shown by perfusion imaging [38,39]. Hence, it is not clear whether PLR would have the same prognostic information if the no-reflow rate was smaller. Finally, limited clinical application of PLR as it is not routinely measured before PPCI.

## 5. Conclusions

High PLR and lower EF are strong, independent predictors of no-reflow in STEMI patients undergoing PPCI. Assessment of PLR might be considered to address patient prognosis and serve as a useful biomarker in the risk stratification model.
